# The Bergen 4-Day OCD Treatment Delivered in a Group Setting: 12-Month Follow-Up

**DOI:** 10.3389/fpsyg.2018.00639

**Published:** 2018-05-03

**Authors:** Bjarne Hansen, Kristen Hagen, Lars-Göran Öst, Stian Solem, Gerd Kvale

**Affiliations:** ^1^OCD-Team, Haukeland University Hospital, Bergen, Norway; ^2^Department of Clinical Psychology, University of Bergen, Bergen, Norway; ^3^OCD-Team, Molde Hospital, Molde, Norway; ^4^Department of Psychology, Stockholm University, Stockholm, Sweden; ^5^Department of Psychology, Norwegian University of Science and Technology, Trondheim, Norway

**Keywords:** OCD, ERP, concentrated exposure treatment, Bergen 4-day program, group format, long term follow-up, patients' acceptance

## Abstract

The Bergen 4-day concentrated exposure treatment (cET) for obsessive-compulsive disorder (OCD) has proven highly acceptable; with practically no drop-out and a 6 month remission rate of nearly 70%. The aim of the present study was to evaluate long term gains of the approach, and to compare the results to findings from our recent meta-analysis. Sixty-nine of 95 patients consecutively referred to an outpatient clinic in the specialist health care, were offered the Bergen 4-day treatment. Among the 65 who initiated treatment, 60.0% were classified with “severe” to “extreme” OCD. None of the patients dropped-out during treatment. Independent Yale-Brown Obsessive-Compulsive Scale interviews were conducted post-treatment, and at 3- and 12-month follow-up. Using the international consensus criteria, 83.1% responded to treatment at 12-month follow-up, and 67.7% of patients were classified as recovered. Significant changes were also seen in depression, as measured by Patient Health Questionnaire-9, and in generalized anxiety, as measured by Generalized Anxiety Disorder-7 scale. A total of 89% of the patients rated the treatment as very good and 100% would recommend the treatment to a friend. Compared to results in a recent meta-analysis, the Bergen 4-day treatment is favorable in respect to attrition, response and 12-month recovery. In sum the Bergen 4-day treatment is a feasible way to deliver treatment for OCD, and the effects are stable at 12-month follow-up. Implications for dissemination are discussed.

## Introduction

Obsessive-compulsive disorder (OCD) has an estimated lifetime prevalence of approximately 2% and the majority of patients are affected before their mid-twenties (Kessler et al., 2005). OCD has been ranked by the WHO among the 10 most debilitating disorders (World Health Organization, 1999). Untreated the disorder tends to be chronic (Koran et al., 1996). This implies that each patient who is not helped represents vast personal- and socioeconomic costs. The recommended treatment of choice for OCD is cognitive behavioral therapy (CBT; National Collaborating Centre for Mental Health, 2006), which refers to exposure and response prevention (ERP) with or without the inclusion of cognitive therapy strategies. Despite documented effective, <50% of the OCD-patients can expect long-term recovery after CBT-treatment (Abramowitz, 1998; Olatunji et al., 2013; Öst et al., 2015).

We^1^ have recently developed a new treatment format, the Bergen 4-day treatment, for patients suffering from OCD, where exposure treatment delivered during four consecutive days. Compared to a recently published a meta-analysis of 37 controlled studies of cognitive behavior therapy (CBT) for OCD in adults, all using Yale-Brown Obsessive Compulsive Scale (Y-BOCS; Goodman et al., 1989a,b) as primary outcome measure (Öst et al., 2015) the results from the 4-day treatment are good. Before treatment the patients receiving the 4-day format were as severely affected by OCD as the patients in the meta-analysis, but after treatment the patients in the 4-day format had significantly less OCD symptoms as compared to patients who had received standard exposure and response prevention (ERP) (*t* = 4.33, *p* < 0.0001). Pooled results from our previous studies show that at post-treatment 86% of the patients have a clinically significant response, and 6 months later 68% are in remission (Havnen et al., 2013, 2017; Öst et al., 2015). The proportion of responders in the meta-analysis was 67% at post-treatment, and 65% at 3- and 12-month follow-up, whereas proportion of remitters was 50, 45, and 49%, respectively. Thus, it seems as cET might be as effective as standard ERP (if not more effective) at post-treatment, and the effects seem to be maintained at a short-term follow-up.

The dropout rate for cET was only 1.3% (Havnen et al., 2014, 2017). This is remarkably low as compared to the overall dropout rate of 19.7% reported in a meta-analysis of Swift and Greenberg (2012) covering 669 RCTs and almost 84,000 patients. In our previous studies of the 4-day treatment the refusal rate was 2.5%. Dropout rate for ERP has been found to be approximately 15% across studies and similar to other treatments for OCD (Ong et al., 2016).

The cET can be considered as “individual treatment in a group setting,” since it is delivered in a group of 3–6 patients with the same number of therapists. The 1:1 ratio between patients and therapists allows for individually tailored and therapist assisted exposures in numerous OCD-relevant settings, and simultaneously the format provides the patients with an opportunity to observe and follow others going through parallel processes. Since the concentrated exposure treatment (cET) is delivered during 4 consecutive days, it is highly attractive to a substantial number of patients, since they during a very short time may get rid of a severe problem which highly impairs everyday life. This is also reflected in the low declining rate (2.5%) and low drop-out rate (1.3%) in the two Havnen et al. (2014, 2017) studies. Also, the format yields an opportunity for OCD-specialists within a given geographical area to join efforts and work together with severe patients during clearly specified time-slots.

One of the strengths of the 4-day format is that it has been developed within an ordinary specialist health care in a clinic responsible for delivering care to all OCD patients in a catchment area of 440,000, which makes the ecological relevance high.

However, results from other brief interventions indicate that even though they might yield good post-treatment results, there could be some deterioration at short-term follow-up (Jónsson et al., 2015), which makes it highly important to evaluate long-term effects in the Bergen 4-day format. The Bergen 4-day treatment has received substantial interest^2^ and the aim of the present study was to evaluate the short- and long-term effects in a new sample of OCD-patients. We also wanted to evaluate the patients' acceptance of the treatment as well as attrition, and to compare the results from the 4-day treatment with the long term results reported in a recent meta-analysis (Öst et al., 2015). Based on the 6-month follow-up results in a previous studies (Havnen et al., 2013, 2014, 2017), as well as the 12-month follow-up data of standard ERP described above, we expected that the effects of cET at post-treatment will be maintained at the 12-monthfollow-up, also compared to the results from CBT as reflected in the meta-analysis.

## Methods

### Referrals

**Figure 1:**
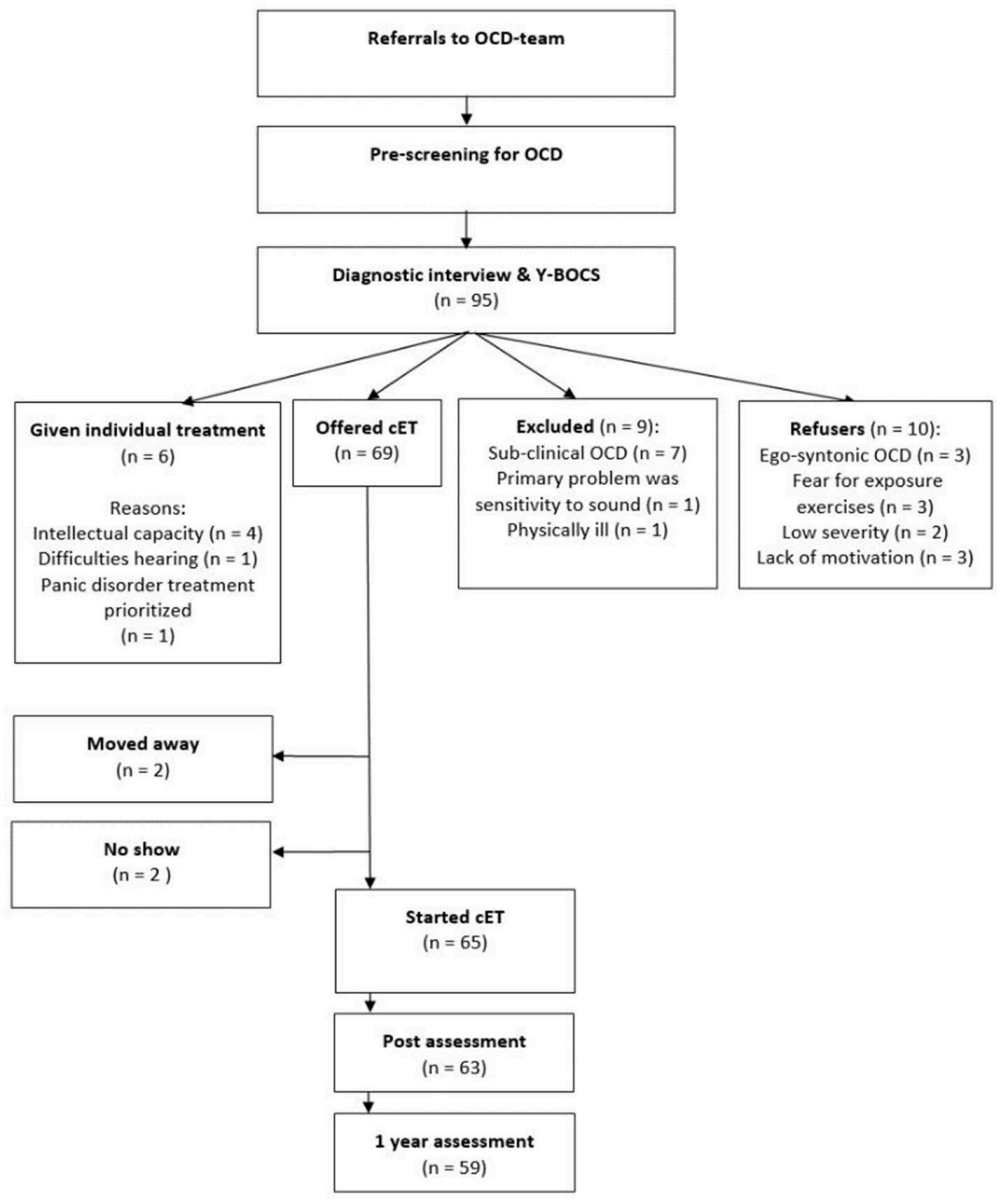
Flow chart.

The data presented are part of a standard quality control performed at the OCD treatment outpatient unit at Haukeland University hospital, which is part of the ordinary specialist health care. Patients are first referred from their general practitioner to a local outpatient clinic, and if the patient is considered to suffer from OCD with a severity that entitles them to care in the specialist health care, the outpatient clinics refer the patient to the OCD-team who does the full screening and diagnostics of the patient. Because the OCD-team is part of the secondary mental health services, all patients fulfilling a principal DSM-IV diagnosis of OCD (American Psychiatric Association, 1994) according to the administration of the Mini International Neuropsychiatric Interview (MINI; Sheehan et al., 1998) have to be offered treatment. Treatment is, however, not initiated if the patient is suicidal, psychotic or in active substance abuse. Furthermore, treatment in a group format is not offered if the patient does not speak Norwegian. Between September 2015 and July 2016, 95 of the patients referred to the clinic fulfilled diagnostic criteria of OCD (for overview, see Figure 1). Ten patients declined any treatment for the following reasons: the OCD was ego-syntonic (3), fear for exposure exercises (3), low severity when they met for screening (2), and lack of motivation (2).

Of the 95 referrals during the specified time period, nine patients were not offered treatment due to following reasons: having a sub-clinical OCD (7), primarily suffering from sensitivity to sound (1), and severe physical illness (1). Six patients were offered individual exposure and response prevention, due to the following reasons: intellectually challenged (4), severe hearing problems (1), and treatment of a panic disorder was prioritized (1), and this treatment was not completed in the specified time period. Figure 1 presents a flow-chart and includes the four sub-groups of patients (not offered treatment; given individual treatment; declined any treatment; offered 4-day treatment). In line with what is expected in the specialist health care, patients with the lesser OCD-severity was not offered treatment: There was a significant difference between the groups on Y-BOCS pre-treatment, *F*_(3)_ = 15.21, *p* < 0.001, and *post-hoc* test using Tukey HSD showed that the significant difference was between the patients not offered treatment (mean Y-BOCS of 15.1, *SD* = 5.7) and the other three groups. Thus, the patients not offered treatment had lower Y-BOCS scores, indicating less OCD-severity, than patients who were offered treatment, refused treatment, or were given individual treatment (*p* < 0.001). There were no significant differences on Y-BOCS between patients who were offered treatment and patients who were given individual treatment or those who declined treatment. No significant difference was found between the groups on GAD-7 [*F*_(3)_ = 1.12, *p* = 0.347], or PHQ-9, [*F*_(3)_ = 2.07, *p* = 0.11].

Furthermore, there were no significant differences between the groups with respect to number of comorbid disorders, *F*_(3)_ = 1.43, *p* = 0.238, age, *F*_(3)_ = 0.75, *p* = 0.53, gender, $\chi_{\left(3\right)}^{2}$ = 2.85, *p* = 0.42, work status (working/studying vs. other), $\chi_{\left(3\right)}^{2}=$ 3.16, *p* = 0.37, or marital status, $\chi_{\left(3\right)}^{2}$ = 5.58, *p* = 0.13.

### Patients in the 4-day treatment

A total of 69 patients were thus offered, and accepted the Bergen 4-day treatment. However, two of these moved to another health region before the treatment started, and another two were prohibited from starting due to physical health issues. Thus, 65 patients initiated treatment, and no patients dropped out after starting treatment.

#### Age, sex, employment, marital status, and education

The patients had a mean age of 32.10 (*SD* = 12.94) and 70.8% were women. A total of 32% were working, 31% were studying, whereas 37% received different social benefits. A total of 51.6% were single. With respect to education: 19 had completed high school, 32.3% had a college degree, while the remaining reported other types of education.

#### OCD severity

Mean pre-treatment Y-BOCS score was 25.83 (*SD* = 4.65). A total of 39.4% had a moderate OCD (Y-BOCS of 18–23), and 60.6% had severe OCD (Y-BOCS of 24-38). The mean Y-BOCS pre-treatment scores for treatment naïve patients (*M* = 28.33, *SD* = 7.65) and patients with previous therapy trials (*M* = 26.11, *SD* = 4.14) did not differ significantly, *t*_(42)_ = 1.19, *p* = 0.24.

#### Comorbidity

Patients had a range of 0 to 4 comorbid disorders. A total of 26 patients (40%) were diagnosed with OCD only, whereas 19 patients had one comorbid disorder, 14 had two, five had three coexisting disorders, and one patient had four comorbid disorders. 17 patients fulfilled the criteria of generalized anxiety disorder (GAD), 17 of depression, 12 of panic disorder (PD), eight had social anxiety disorder, and three patients fulfilled the criteria of post-traumatic stress disorder (PTSD). Other comorbid disorders included specific phobia, bulimia, anorexia, health anxiety, and trichotillomania and ADHD as reported in the referrals.

#### Pharmacological treatment

Use of medication was registered at the initial interview. A total of 46.2% (*n* = 30) were using psychotropic medication. More specifically, 41.5% used SRI/SSRI/SNRI, 7.7% used benzodiazepines, 10.8% used anti-psychotics, and 4.6% used Ritalin. Patients with and without psychotropics did not differ on Y-BOCS scores [*t*_(63)_ = 0.14, *p* = 0.89], PHQ-9 scores [*t*_(64)_ = 0.19 *p* = 0.85], or GAD-7 scores [*t*_(64)_ = 1.01, *p* = 0.32] at pre-treatment. Patients on SSRI were encouraged to keep medication doses unchanged prior to and during the 4-day treatment period. Patients with prescribed anxiolytics were asked to discontinue these medications prior to and during treatment; adherence to these instructions was urged by the therapists throughout the treatment period. Patients were asked not to seek concurrent treatment during the treatment period.

### Procedure

#### Assessment

All patients were informed that quality control procedures were an integrated part of the 4-day treatment, and all referred patients were asked to complete a number of questionnaires administrated online (for description, see below) prior to the first visit at the clinic. For the patients who were offered the Bergen 4-day treatment these questionnaires were also completed post-treatment (conducted 1 week after the 4 days of therapy), as well as at 3-and 12-month follow-up.

The MINI interviews were conducted by a psychiatrist or a clinical psychologist trained in the administration of this structured clinical interview. The Y-BOCS interviews at pre-treatment were conducted by one of the psychologists or psychiatrists in the OCD-team. Y-BOCS at post-treatment, as well as at 3- and 12-month follow-up, were conducted over the phone by a specially trained independent psychologist. The assessors were aware that the patients had received concentrated ERP treatment, but were not involved in the treatment. At 12-month follow up, 20% of the sample was randomly selected to be re-interviewed within 1 week by another independent psychologist. The inter-rater reliability, Intra Class Coefficient (3. 1) = 0.95, was excellent. The independent assessors were aware that the patients had received concentrated ERP treatment, but were not involved in the treatment.

Referred patients met a clinician for an initial screening session and if an OCD-diagnosis was indicated, they met for one to two additional diagnostic sessions where anamnestic information was obtained, a MINI and a Y-BOCS were performed and the outline of the treatment presented.

#### Preparation for treatment

In order to ensure standardized information regarding the Bergen 4-day format, patients watched a video presenting the outline of the treatment https://www.youtube.com/watch?v=nqx8knpy3i4&feature=youtu.be After watching the video, the patients' expectations of treatment outcome as well as their evaluation of the treatment credibility, was assessed with an adapted version of the Borkovec and Nau (1972) *Reaction to treatment scale*, in which four aspects of expectancy and credibility were evaluated on a 0–100% scale, with higher values indicating more positive evaluations. If a patient reported an expectancy- or credibility score below 70%, this was taken as an opportunity to clarify possible misunderstandings regarding the treatment. When the patients were offered participation in a group, and accepted this, they were informed in detail about the 4-day treatment, and in order to ensure that this was conducted in a standardized way, the patients watched the following video: https://www.youtube.com/watch?v=1Fnxt0_ljpY&feature=youtu.be. As preparation for the group the patients were instructed to suggest relevant exposure tasks, and as guidance they were told that the exposure tasks “that their OCD would appreciate the least” often were the most relevant.

### Treatment

Treatment was delivered as part of the standard mental health care provided by the OCD team and it was conducted in groups of 3–6 participants with a 1:1 therapist-patient ratio. This means that each therapist was able to deliver full treatment for one patient during less than a week. A full manual has been developed and is currently being translated to Icelandic and to Swedish (Kvale and Hansen, unpublished manuscript).

The first day (approximately 3 h) was allocated to psychoeducation and, in the group setting, to prepare individual exposure taks. The two middle days were dedicated to individually tailored and therapist assisted exposure training (8–10 h each day) in a wide range of OCD-relevant settings, and the last day to summarize “lessons learnt” and preparing for the next 3 weeks of self-administered exposure training. Relatives and friends were invited to a psycho-educative meeting in the afternoon of day 3.

The main feature of our 4-day intervention is to teach the patients to approach whatever elicits the relevant anxiety or discomfort, and to help them systematically learn how to “**LE**an into **T**he anxiety” (LET- technique) instead of employing obvious or subtle avoidance. This is done in numerous relevant situations with a therapist as a coach. During two consecutive days the therapists assist each patient to practice the LET-technique consistently whenever anxiety or discomfort is elicited, and he patient is encouraged to approach as many anxiety- or discomfort eliciting situations, contexts, and thoughts as possible. To ensure that therapist assisted exposure training to the most context-relevant cues and situations are included, it is a prerequisite that the patient live within a 1 h travel distance to the clinic. On the last of the 4 days, strategies for maintaining the change and principles for how to be their own therapist were focused. The next 3 weeks, the patients were encouraged to report online every day on how they were practicing the cET. The clinicians read the reports, but there was no contact with the patients. On day 4 of the treatment the patients were informed orally and in writing how to contact the health care if an emergency situation should occur.

Three months after treatment, patients were invited to an individual session (0.5 h) where their experiences in the period following treatment were discussed. The principles of cET were repeated and emphasis was put on how the patients' best could practice the method on their own. No exposure work was conducted in this session.

### Therapists

All the 4-day treatment groups were led by experts on the format. In order to qualify as a cET-expert, therapists must, in addition to having extensive training and experience as an OCD-therapist, have participated and received supervision in a minimum of two cET groups and demonstrate competency in the exposure procedure in accordance with a slightly modified version of the OCD CORE competencies (Steketee, 1993) evaluated independently by two cET experts. The group leader had always participated in a minimum of 6 groups as a cET expert.

### Measures

*Yale-Brown Obsessive Compulsive Scale* (*Y-BOCS*; Goodman et al., 1989a,b) interview consists of 10 items covering the severity of both obsessions and compulsions, and is frequently used to assess OCD severity. The Y-BOCS has good psychometric properties (Goodman et al., 1989a,b).

*Patient Health Questionnaire-9* (*PHQ-9*; Kroenke et al., 2010) is a self-administered screening instrument containing 9 questions each ranging from 0 to 3, yielding a maximum score of 27. According to Kroenke et al. (2010), a score of 10 or more is indicative of a depressive disorder. The psychometric properties of PHQ-9 are well-established (Titov et al., 2011; Feng et al., 2016).

*Generalized Anxiety Disorder Scale* (GAD-7; Spitzer et al., 2006) measures symptoms of generalized anxiety. The psychometric properties are well-established (Kroenke et al., 2010; Beard and Bjorgvinsson, 2014; Hinz et al., 2017; Rutter and Brown, 2017).

*Client Satisfaction Questionnaire 8* (CSQ-8; Nguyen et al., 1983). The CSQ-8 is an 8-item questionnaire which measures patient satisfaction with health services. Each item is scored from 1 (very low satisfaction) to 4 (very high satisfaction), total score ranges from 8 to 32. The CSQ-8 has sound psychometric properties (Larsen et al., 1979; Nguyen et al., 1983).

### Treatment response

Severity of OCD was assessed at pre, post, 3- and 12-month follow-up by specially trained raters. Treatment response was calculated based on the *international consensus criteria* (Mataix-Cols et al., 2016) which requires a ≥ 35% reduction of the individual patient's pre-treatment Y-BOCS score in order to be classified as a clinically relevant response. A patient is classified as remitted if the post-treatment Y-BOCS score is ≤ 12 points. Recovery is defined by meeting the remission criterion at 12-month follow-up.

We also used the Jacobson and Truax (1991) criteria for *clinically significant change*. First, response is defined as reliable change index (RCI) which requires that the individual patient's change from pre to post (follow-up) must be large enough to be statistically reliable at the *p*-level of 0.05. In this sample the RCI was 8 points. Second, recovery is defined as fulfilling RCI and cutoff C; the mean between the patient sample and a normal sample. For this analysis we used as normal mean the weighted mean of the samples published by Dannon et al. (2006) and Johansen and Dittrich (2013), and this resulted in a cutoff score of 11.

### Statistical analyses

Statistical analyses were performed with SPSS version 24.0. Repeated measures ANOVA for Y-BOCS, GAD-7, and PHQ-9 were conducted with Greenhouse-Geisser corrections. Effect sizes were calculated with Cohen's *d*, defined as (M_pre_-M_post_)/SD_pre_ as recommended by Morris and DeShon (2002). There were no missing data for Y-BOCS or GAD-7 pre-treatment. A total of 63 patients were available for Y-BOCS evaluation post-treatment, 54 at 3-month and 59 at 12-month follow up. Four patients had a missing GAD-7 score post-treatment, and 19 at 3-month follow-up. One patient had a missing PHQ-9 score pre-treatment and 19 at 3-month follow-up. A total of 10 patients had missing values on CSQ-8 at post-treatment. Missing data were replaced using the expectation-maximization method of SPSS, version 24. The method was chosen to allow for repeated measures ANOVA. Effect estimates were also obtained by estimating both a random intercept model and a random slope model, taking into account potential clustering at treatment group level as well as at the individual patient level. Likelihood ratio test was applied to compare these two models, and the random intercept model was chosen since there was no gain in allowing for random slopes. ICC was calculated from this random intercept model.

## Results

### Declining treatment and attrition

There were 10 patients (12.7%) who fulfilled inclusion criteria but declined the offer of receiving the 4-day treatment (see Method for description of reasons). All patients who started the treatment also completed it so the attrition rate was 0%.

### OCD-symptoms

Table 1 displays the results for Y-BOCS pre-treatment, post-treatment, 3-month and 12-month follow up. Repeated measures ANOVA (Wilks' Lambda) found a significant effect of time, *F*_(3, 62)_ = 182.95, *p* < 0.001, $h_{p}^{2}$ = 0.90. There were no significant changes in symptoms from post-treatment to follow-up assessment at 3- and 12-month (*p* = 0.76 and *p* = 0.82). Table 2 shows the coefficient estimates from the chosen random intercept model. This model also indicated a significant effect of time (*p* < 0.001) (see Table 2). The ICC from this model was 0.017 on group level and 0.09 on the individual level, indicating that clustering at treatment group level is small, while there are only moderate correlations between measures within each patient.

**Table 1:** Means, standard deviations and effect sizes (Cohen's *d*) for Y-BOCS, GAD-7, and PHQ-9.

***Y-BOCS***	***M***	***SD***	***d***
Pre	25.83	4.65	
Post	10.24	5.13	3.35
3-month	10.45	5.86	3.31
12-month	10.64	7.00	3.27
***GAD-7***			
Pre	11.51	5.15	
Post	9.29	4.91	0.45
3-month	8.36	5.09	0.61
***PHQ-9***			
Pre	11.54	5.93	
Post	9.17	6.17	0.40
3-month	9.18	5.81	0.40

*N = 65 (treatment starters)*.

**Table 2:** Random interception and random time slope linear mixed model.

	***b***	***SE_*b*_***	**95% *CI***
Constant	21.08	0.80	19.52, 22.64
Time	−4.54	0.39	−5.30, −3,77

### Clinically significant change in OCD-symptoms

To determine the number of patients who showed clinically significant change we applied the international consensus criteria for clinical improvement. At post-treatment 93.8% of the patients had responded and 76.9% were in remission. At 3-month follow up, 81.5% responded, 72.3% were in remission, and at 12-month follow-up 83.1% were classified as responders and 67.7% as recovered. When using the asymptomatic criterion on Y-BOCS (≤7), 19 patients (29.2%) at post-treatment and 25 (38.5%) at 12-month follow-up were considered asymptomatic.

Clinically significant change was also calculated in according to the Jacobson and Truax (1991) criteria. At post-treatment 93.8% of the patients fulfilled RCI and 67.7% the cutoff for clinically significant change. The corresponding proportion at 12-month follow-up was 86.2 and 64.6%, respectively.

Table 3 shows the clinical improvement at post-treatment and 12-month follow-up for the individual patients, using the international consensus criteria. Of the 50 patients who were in remission at post-treatment, 35 patients (70%) were classified as recovered at follow-up. Of the 11 patients who were classified as responders at post-treatment, seven (64%) had become recovered follow-up, one remained as a responder, whereas three had deteriorated to the category of no change. Of the four patients who were classified as unchanged at post-treatment, two were recovered at follow-up, one had a treatment response, and one remained unchanged.

**Table 3:** Comparison of clinical improvement rates at post-treatment and follow-up.

	**12-month follow-up**			
	**Recovered**	**Responded**	**Unchanged**	**Total**
**POST-TREATMENT**				
Recovered	35	8	7	50
Responded	7	1	3	11
Unchanged	2	1	1	4
Total	44	10	11	65

Clinically significant change as defined by the international consensus criteria was also calculated for the subgroups moderate and severe/extreme OCD severity as assessed at pre-treatment. For the moderate subgroup 80.8% were classified as remitted at post-treatment, 73.1% at 3-month follow-up, and 76.9% as recovered at 12-month follow-up. For the severe/extreme subgroup; 74.4% were remitted at post-treatment, 71.8% at 3-month follow up, and 61.5% were recovered at 12-month follow-up. Treatment response for the two subgroups at 12-month follow-up was not significantly different, $\chi_{\left(1\right)}^{2}$ = 0.19, *p* = 0.28.

### Depression and generalized anxiety

Table 1 displays the results at pre-treatment, post-treatment, 3- and 12-month follow-up for depressive symptoms and generalized anxiety. Repeated measures ANOVA (Wilks' Lambda) showed a significant reduction in anxiety symptoms, *F*_(2, 63)_ = 15.43, *p* < 0.001, η_*p*_^2^ = 0.329. From post-treatment to 3-month follow-up there were no significant change (*p* = 0.08) but tendencies toward further improvement.

Repeated measures ANOVA for depressive symptoms (Greenhouse-Geisser correction) found a significant effect of time, *F*_(1.82, 63)_ = 13.90, *p* < 0.001, $\eta_{p}^{2}$ = 0.178. There were no significant changes from post-treatment to 3-month follow-up (*p* = 0.993).

### Treatment satisfaction

Details about treatment satisfaction are presented in Table 3. Fully 89.2% described the quality of treatment as excellent and 87.7% stated that they were highly satisfied overall with the treatment (see Table 4). Ninety-two percent stated that they were satisfied with the amount of treatment. Ninety-eight percent stated that they would return to the clinic in the future if they needed treatment. Overall, the results from CSQ-8 indicated that the majority of patients were highly satisfied with the concentrated treatment format. There was no significant difference on Y-BOCS post-treatment between patients that completed the CSQ-8 and patients that did not, *t*_(63)_ = 1.76, *p* = 0.08.

**Table 4:** Post-treatment scores on client satisfaction questionnaire 8.

	**Scale Score**			
	**1**	**2**	**3**	**4**
1. Quality of service	0	0	7	58
2. Kind of service	0	2	18	45
3. Met needs	0	3	33	29
4. Recommend to a friend	0	0	5	60
5. Amount of help	4	1	10	50
6. Deal with problems	0	0	9	56
7. Overall satisfaction	0	1	7	57
8. Come back	0	2	7	56

*Mean CSQ score was 29.91 (2.33), range 19–32, mode = 30, median = 30.0 (possible range is 8–32)*.

### Comparison with our previous studies

We combined the Y-BOCS data from our previous studies (Havnen et al., 2014, 2017; *N* = 77) yielding the following means (SD): pre-treatment 25.9 (4.33) and post-treatment 10.0 (4.26). The present sample had at pre score of 25.8 (4.65) and post score of 10.2 (5.13). The differences at pre (*t* = 0.13) and at post (*t* = 0.25) were both non-significant. Thus, the present sample of OCD-patients achieved as good a treatment effect as our previous samples. A tentative comparison of the follow-up results, which was done after 6 months in our previous studies (11.3 ± 6.08) and 12-month in the present study (10.6 ± 7.00), also showed a non-significant difference (*t* = 0.64). Also, in the present study the change between the post mean (10.2) and the follow-up mean (10.6) was not significant.

### Comparison with other studies on ERP for OCD

In order to obtain a perspective of the outcome of the Bergen 4-day treatment (cET) we compared it to standard ERP from randomized controlled trials as described in a recent meta-analysis (Öst et al., 2015). Table 5 shows the outcome for Y-BOCS and proportion achieving the criterion for response, remission, and recovery (at follow-up). There was no significant difference at pre-treatment but at post-treatment and at 12-month follow-up cET had a significantly lower Y-BOCS mean than standard ERP. Furthermore, the within-group effect size for the 4-day treatment was higher than that for standard ERP (*d* = 3.35 vs. 2.38) at post-treatment as well as at follow-up assessment (*d* = 3.27 vs. 2.46)^3^.

**Table 5:** Severity of anxiety and depression at pre-treatment, post-treatment and 3-months follow-up.

	**GAD-7**			**PHQ-9**		
**Severity**	**Pre**	**Post**	**3 m**	**Pre**	**Post**	**3 m**
None	5	6	14	8	18	15
Mild	19	38	30	19	24	23
Moderate	21	10	12	21	11	17
Severe	20	11	9	17	12	10

*Pre, Pre-treatment; Post, Post-treatment; 3 m, 3-months follow-up. Cut-offs used for both GAD-7 and PHQ-9 were 0–4 (none), 5–9 (mild), 10–14 (moderate), 15 and above (severe)*.

Proportion of responders was significantly higher in cET than in standard ERP both at post-treatment (93.8%) and at follow-up (83.1%). More importantly, cET yielded a significantly higher remission rate at post-treatment (76.9%) and a significantly higher recovery rate at follow-up (67.7%) than standard ERP (see Table 6).

**Table 6:** Comparison between the Bergen 4-day treatment and standard ERP.

	**Bergen 4-day**		**Standard ERP**		**Statistic**	
***Y-BOCS***	***N***	***M (SD)***	***N***	***M (SD)***	***t*-test (*p* =)**
Pre	65	25.8 (4.65)	583	25.1 (4.88)	1.10 (0.271)
Post	65	10.2 (5.13)	552	13.5 (6.92)	3.72 (0.0002)
1 year f-up	65	10.6 (7.00)	105	13.1 (8.08)	2.06 (0.041)
**INTERNATIONAL CONSENSUS CRITERIA**						
**Response (**≥ **35% reduction)**	**Fisher 2-tailed**				
Post	65	93.8%	87	57.5%	*p* < 0.0001
1 year f-up	65	83.1%	23	52.2%	*p* = 0.0053
**Remission/recovery (** ≤ **12 on Y-BOCS)**
Post	65	76.9%	101	47.5%	*p* = 0.0002
1 year f-up	65	67.7%	104	44.2%	*p* = 0.0042
**JACOBSON AND TRUAX (****1991****) CRITERIA**						
**Response (Reliable Change Index;** ≥ **8 points' reduction)**	**Fisher 2-tailed**				
Post	65	93.8%	87	57.5%	*p* < 0.0001
1 year f-up	65	86.2%	23	52.2%	*p* = 0.0026
**REMISSION/RECOVERY (** ≤ **11 ON Y-BOCS)**						
Post	65	67.7%	101	47.5%	*p* = 0.0161
1 year f-up	65	64.6%	104	44.2%	*p* = 0.0115

*Data for Standard ERP were taken from randomized controlled studies of ERP in the meta-analysis of Öst et al. (2015). N indicates the total number of participants across these studies. Regarding the international consensus criteria response was calculated for ERP-studies using ≥35% reduction on Y-BOCS. Remission and recovery were calculated for those ERP-studies using ≤ 12 on Y-BOCS (or lower) plus the response criterion*.

## Discussion

We have previously shown in an effectiveness study and a replication study that the Bergen 4-day treatment yields large clinical change in adult patients with OCD and also is highly accepted both as indicated with low refusal and exceptionally low drop-out rates compared to a recently published meta-analysis (Öst et al., 2015). The focus of the current study is to report long-term effects. Based upon our previous findings we expected comparable results in a new sample and that the effects of cET at post-treatment will be on a par with our previous studies (Havnen et al., 2014, 2017) and maintained at the 12-month follow-up.

As expected, both acute and long-term clinical effects were maintained. The patients had the same pre-treatment OCD-severity as the ERP-patients in our recent meta-analysis (Öst et al., 2015), but at post-treatment and at 12-month follow-up the patients receiving the 4-day treatment had improved significantly more than patients treated with standard ERP. This was not only seen in the effect sizes, but the proportions of patients with clinically significant change and the proportion of patients who were remitters. It is also noteworthy that the number of patients with a Y-BOCS score of 7 or below, which is termed asymptomatic, is remarkably high. In a Meta-analysis by Fisher and Wells (2005) they found that 27% of the patients were asymptomatic at post-treatment and that further improvement does not occur during the follow-up period. In our study we found an increase in the number of asymptomatic patients from 29.2% (*n* = 19) at post-treatment to 38.5% (*n* = 25) at 12-month follow-up.

The low refusal rate, and exceptionally low attrition we found in our previous studies, were replicated, with a drop-out of 0. Given that the current results are obtained in an ordinary outpatient clinic which is required to deliver care to all OCD-patients in the catchment area, these results are remarkable, since the mean attrition rate in effectiveness studies (Öst et al., 2015) is 15.1% (*SD* = 12.5, range 0–41%). This result alone, suggest a substantial cost-effectivenes with the 4-day treatment.

Furthermore, the patients are highly satisfied with the quality, relevance and amount of treatment, and all of the patients would recommend the 4-day treatment to a friend with similar problems. This result is also in line with the day-to day experience of demand for the 4-day treatment, both from patients as well as relatives.

As in our previous studies, we also saw a significant effect on depression and on generalized anxiety. However, these kinds of comparisons of non-randomized groups should always be regarded with caution but a tentative conclusion is that the concentrated exposure treatment seems to be at least as effective as standard ERP, if not more effective.

It might be speculated what makes the cET so effective, and the combination of a number of features might be relevant: Firstly, since the treatment is concentrated over four consecutive days where the first is dedicated to preparing the patients for the exposure training and the last one for summarizing and preparing for how to integrate the change into normal every-day living, the two middle days basically works as one long treatment session separated by a night of sleep. Since the treatment is delivered in groups of 3–6 patients with the same number of therapists, this allows for individually tailored and therapist assisted exposures in a wide range of relevant setting. Simultaneously, the patients can take advantage of being together with others going through basically the same process of change. Also, the focus on “how-to-do” the exposures without simultaneously applying subtle avoidance strategies, is an important feature of the cET. Anxiety and discomfort is labeled the raw material for change, and the task is to approach as many potentially valuable situations as possible, and pay attention to not holding back while doing the exposures, but rather do something that is incompatible with the OCD. This might for examples imply that the patient choose not merely to refrain from rituals (e.g., washing) or from various reassurances (“is this safe to do”) but to actively increase the uncertainty during a given exposure task e.g., by reminding themselves that they cannot be certain that they were not contaminated with something harmful. This approach might facilitate robust change, since the patients basically learn a new approach to regulate anxiety and discomfort, meaning that recurrence of anxiety is just to be regarded as a new opportunity to practice the “leaning in” technique.

In sum, the 4-day treatment seems to be a highly feasible approach to treating patients with OCD. The acceptance is high, the drop-out is low and the clinical change obtained seems to be superior to what is typically seen in standard ERP. The patients want the treatment, and all of them would recommend it to a friend with similar problems. Furthermore, the approach represents a highly flexible and at the same time predictable use of resources with a clearly defined start and completion of the treatment. Since the 4-day approach is developed within an ordinary outpatient clinic, the relevance of the approach is undisputable. However, the results obtained from one clinic only, and decline of quality and loss of effects might occur when a new treatment approach is disseminated (Johnsen and Friborg, 2015).

There is a high demand for the Bergen 4-day treatment both from patients, relatives as well as health care institutions, and when we now start to disseminate this new treatment, we have decided upon an approach that aims at ensuring that the quality is maintained. Basically this is done by a combination of efforts that enables bench-marking between clinics. Firstly, the Bergen 4-day treatment is only to be offered by clinics who have agreed to train and certify a minimum of three 4-day therapists, one of which is certified as a group leader, and to deliver the treatment in accordance with all parts of the manual and procedures. Since the treatment is delivered during 4 consecutive days to groups of 3–6 patients with the same number of therapists, it provides an excellent arena for hands on supervision where a junior therapist can learn by observing and working side by side an experienced therapist. Also, this means that the certified therapists directly observe the trainees and ensure that they are following the described and established procedures. Before a given therapist is granted hands-on supervision and training, they have to attend to a basic introductory course presenting the essentials of the treatment. We have also developed brief courses for group leaders and for administrative staff. When a new clinic sets up a 4-day team, this is done in close collaboration with our clinic, and certified therapists will participate in the groups the first times it is delivered at a new site, and also evaluate and certify the group leader when performing at the new site. Furthermore, detailed quality assessment is an integrated part of the treatment, and a new 4-day site is required to use the exact same procedure. This enables bench-marking of results and if the results are not within the expected range, the team will get more supervision, evaluation and a new certification procedure. The agreements of collaboration also ensure that a given clinic delivers a minimum of six groups after it is certified, and also requires that it, as long as it delivers the 4-day format, follows the described procedures. All parts of this dissemination approach are done on a self-cost basis.

## Ethics statement

This paper uses data collected as part of the standard assessment procedure at the outpatient OCD-clinic in Helse Bergen, Norway. The study was approved by the Data Protection OfficialAugust 5, 2012.

## Author contributions

BH and GK contributed to the study design; BH, GK, and KH contributed to the data collection. All authors contributed to the statistical analysis, interpretation of the data, and drafting of the manuscript. All authors approved the final version.

### Conflict of interest statement

The authors declare that the research was conducted in the absence of any commercial or financial relationships that could be construed as a potential conflict of interest.

## Footnotes

^1^Gerd Kvale and Bjarne Hansen.

^2^At the moment more than 30 teams in Norway, Sweden and Iceland are trained to deliver the Bergen 4-day treatment.

^3^Since the cET category only has one study the effect sizes cannot be statistically compared.
